# Correction: NK cell–derived GM-CSF potentiates inflammatory arthritis and is negatively regulated by CIS

**DOI:** 10.1084/jem.2019142103192020c

**Published:** 2020-03-27

**Authors:** Cynthia Louis, Fernando Souza-Fonseca-Guimaraes, Yuyan Yang, Damian D’Silva, Tobias Kratina, Laura Dagley, Soroor Hediyeh-Zadeh, Jai Rautela, Seth Lucian Masters, Melissa J. Davis, Jeffrey J. Babon, Bogoljub Ciric, Eric Vivier, Warren S. Alexander, Nicholas D. Huntington, Ian P. Wicks

Vol. 217, No. 5, May 4, 2020. 10.1084/jem.20191421.

*JEM* regrets that in the original version of this paper, Fernando Souza-Fonseca-Guimaraes’s name was mistakenly written as “Fernando Guimaraes.” The corrected author list appears above.

